# Emotions and Ethical Decision-Making in Animal Ethics Committees

**DOI:** 10.3390/ani8100181

**Published:** 2018-10-17

**Authors:** Elisabeth Tjärnström, Elin M. Weber, Jan Hultgren, Helena Röcklinsberg

**Affiliations:** 1 Department of Animal Environment and Health, Swedish University of Agricultural Sciences, Box 7068, 750 07 Uppsala, Sweden; elisabeth.tj@mail.com; 2 Department of Animal Environment and Health, Swedish University of Agricultural Sciences, Box 234, 532 23 Skara, Sweden; elin.weber@slu.se (E.M.W.); jan.hultgren@slu.se (J.H.)

**Keywords:** animal ethics committee (AEC), empathy, harm–benefit, laboratory animal welfare, ethical decision-making

## Abstract

**Simple Summary:**

In the EU, research projects using animals must be evaluated and approved by an ethical committee prior to start to balance potential harm to the animals with potential benefit to humans, in order to ensure moral standards, scientific validity, and public trust. However, different levels of knowledge among committee members, different views on which ethical aspects are relevant, member hierarchies, and a discrepancy between prevailing scientific norms of objectivity and the necessary conditions of a proper ethical evaluation makes it challenging. If applications are not properly evaluated, this can cause distrust in the ethics committees by society. We analyzed the role of scientific norms among Swedish committee members, application of the harm–benefit model, and the role of emotions in the ethical decision-making process. Researchers and chairpersons were most positive, whereas laypersons from animal welfare organizations were most negative. Laypersons more often felt emotionally engaged in the evaluation, but also that they felt they had less influence. We argue that the prevailing scientific norms are preventing necessary conditions for sound ethical evaluation consideration by excluding some members from the discourse. We propose that alternative models for ethical decision-making could contribute to an improved process and hence meet public trust.

**Abstract:**

Ethical evaluation of projects involving animal testing is mandatory within the EU and other countries. However, the evaluation process has been subject to criticism, e.g., that the committees are not balanced or democratic enough and that the utilitarian weighting of harm and benefit that is normally prescribed is difficult to carry out in practice. In this study, members of Swedish Animal Ethics Committees (AECs) completed a survey aiming to further investigate the decision-making process. We found that researchers and animal laypersons make significantly different ethical judgments, and hold disparate views on which ethical aspects are the most relevant. Researchers were significantly more content than laypersons with the functioning of the committees, indicating that the ethical model used suited their preferences better. We argue that in order to secure a democratic and proper ethical evaluation, the expectations of a scientific discourse must be acknowledged, while giving room for different viewpoints. Further, to fulfil the purpose of the project evaluations and meet public concern, the functions of the different AEC member categories need to be clarified. We suggest that one way of achieving a more thorough, balanced and inclusive ethical evaluation is to allow for more than one model of ethical reasoning.

## 1. Introduction

The use of animals in research has always evoked ethical dilemmas. These have been more or less acknowledged throughout history, and a variety of views have existed, regarding for instance science, attitudes towards animals, and societal norms [1,2]. Ethical awareness and commitment regarding the use of animals have however grown in the past decades [3], not least regarding animal research [4,5]. In the EU, a so-called project evaluation of research projects involving animals, including an ethical analysis, became mandatory in January 2013 through Directive 2010/63/EU [6]. The Directive states that “It is also essential, both on moral and scientific grounds, to ensure that each use of an animal is carefully evaluated as to the scientific or educational validity, usefulness and relevance of the expected result of that use. The likely harm to the animal should be balanced against the expected benefits of the project. Therefore, an impartial project evaluation independent of those involved in the study should be carried out as part of the authorisation process of projects involving the use of live animals” [6] (Recital 39).

Throughout this article, this process will be referred to as “ethical evaluation” for simplicity, and the committees responsible for the ‘impartial project evaluation’ as Animal Ethics Committees (AECs).

In Sweden the AECs were fully established in 1979, although the first Swedish animal welfare law was enacted in 1945. In 1989 layperson representation was strengthened by prescribing equal numbers of laypersons and science representatives. The idea behind including a high number of lay persons was to establish societal trust and insight in animal-based research ensuring that the ‘view of the society’ was considered in the ethical evaluation [1,7]. Further, a “neutral” or “impartial” chairperson and vice chairperson (both lawyers) were prescribed [8], establishing the current AEC the committee structure. According to Hagelin, et al. [9] the AECs in Sweden have contributed to improving the quality of studies involving animals, e.g., by introducing experimental standards and animal welfare practices. 

**Figure 1:**
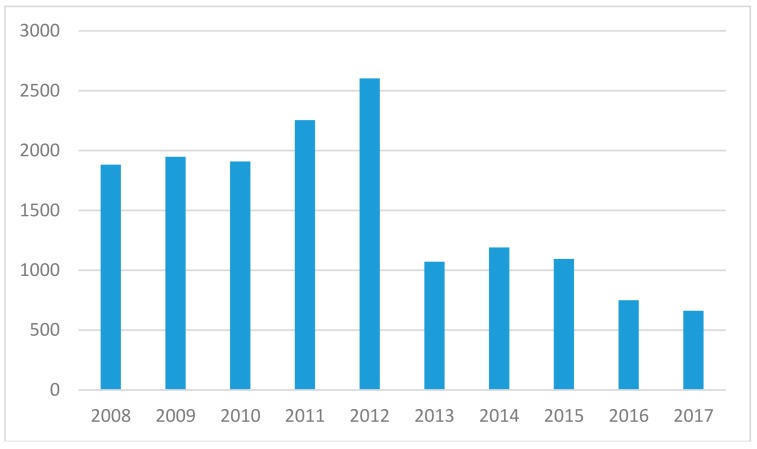
Number of applications sent to Swedish AECs from 2008 to 2017, showing a marked decline from 2013 when application costs increased.

Today there are six AECs in Sweden, linked to the District Courts in all university cities with medical faculties [10]. The AECs are responsible for reviewing all planned research projects at companies or universities involving animal use, regardless of the level of animal suffering caused by the projects. The committees have several functions, one being to secure the credibility of research and ensure scientific quality as well as to scrutinize moral justification e.g., [11]. The review process also enables researchers to reflect upon their own work and avoid becoming blind to possible ethical issues [12]. Animals included in the mandatory review in Sweden are mammals, birds, reptiles, amphibians, fish, and cyclostomes [13]. The Swedish Board of Agriculture is the responsible authority but each committee is an independent decision-making body. The AECs generally meet every month to process applications in panels with about three to six members who make a preliminary decision. The projects are then presented and discussed in a plenary session, where voting and decision-making is carried out. In 2013–2017, an average of 954 applications were submitted [14,15], compared to 2119 applications in 2008–2012 [16,17] (Figure 1). The decrease after 2013 is likely related to an application cost (6000 SEK per application) introduced by the government at the same time as the new EU Directive came into force 2013, resulting in fewer but more extensive applications. There were however no substantial changes in the content of the legislation, since Sweden already had a stricter legislation than the introduced EU Directive.

The AECs consist of one chairperson and a deputy chairperson (both lawyers), six scientific representatives (normally researchers or animal technicians) and six laypersons (four representatives of political parties and two from animal welfare or animal rights organizations) [18]. All members have personal deputies, and one laboratory animal veterinarian and one animal welfare inspector are allowed to participate as advisers during plenary. In principle, the general idea is that AEC decisions shall mirror different societal concerns through the two member categories: researchers are to ensure quality in research design and process, lay persons are to ensure animal welfare, and together they are responsible for safeguarding the overall societal relevance. This opens for a range of challenges of both ethical and procedural character due to sometimes wide differences between the member categories in education, experiences and understanding of what is considered the ethical issue at stake [19,20,21].

The following literature overview highlights ethically relevant issues in international studies of different kinds of AECs, as well as the challenges of combining moral and scientific perspectives and dimensions in the ethical decision-making. After a short description of the moral practice of the AECs, the role of moral emotion in ethical decision-making is highlighted, and finally we elaborate on the discrepancy between scientific norms and moral emotions.

### 1.1. The Moral Practice of AECs

The task of the AEC is to ensure good moral practice, i.e., a decision-making process which ensures that all research involving animals is ethically and scientifically justifiable. Because the public generally puts a lot of trust in the AECs and their evaluation, it is crucial to make sure that the procedure is sound [22]. According to Herzog [23], ethical judgments are “inextricably bound in a complex matrix of emotions, logic and self-interest”. Therefore, better knowledge of the psychology behind human moral reasoning and decision-making is of great importance when aiming at improved animal welfare and virtually any progress in the field of animal welfare [23]. 

Varga [24] concluded that AECs differ in a number of ways, e.g., in organizational structure, approach to decision-making and time constraints. She shows that decisions are often made inconsistently, and risk ignoring ethical aspects of proposed projects. In addition, there is currently a lack of studies about the quality of the outcomes of the ethical reviews [24]. As an example, Plous and Herzog [25], found low levels of consensus on protocol reviews, both within and between committees. Ideland [20] and Schuppli [26] both reported that the harm–benefit analysis is carried out inconsistently, as there are different views of which benefits justify which harms.

Further, Schuppli [26] describes a problem with the AEC assessments being perceived as objective, although neither harms nor benefits, which are central to the ethical model of analysis most commonly used, are really quantifiable or comparable. There is also the view, as expressed by Hills [27], that this utilitarian model would only work if all animals were originally seen as worthy of equal consideration, which is not the case. The efficacy of this utilitarian harm–benefit model of analysis has also been questioned, in that it is by some thought to be very different from moral evaluations in other settings [28]. For example, Schuppli [26] found that in Canadian AECs, considerations such as empathy with animals and attempts to be consistent with previous decisions affected the evaluation. Her interpretation was that the Canadian policy reflected a utilitarian approach to ethics that might come naturally to researchers, but that may poorly reflect how ethical frameworks are used by other committee members to make moral decisions. She therefore suggested that the basis of the policy-mandated harm–benefit assessment should be clarified, and that other ethical frameworks should be included in the guidelines as a basis for proper ethical assessment. 

As pointed out by for example Rollin [22], an important aim and effect of the animal ethics committees has been to legitimize the ethical conflicts surrounding research on animals and to provide a forum to discuss the moral dilemmas involved. However, the dependence of the committees on the scientific community is another aspect of AEC work that has been criticized and questioned, not least by Forsman [11], who states that it is very difficult to “criticize the (scientific) system from within”. In many committees, scientist members comprise the majority, and Plous and Herzog [25] concluded that it can be problematic to have membership composition in the committees skewed towards these, as they have vested interests in continuing animal research. 

Forsman [11] concluded that committee members are easily defined as either “in or out of the system”, which may lead to a problem with committee democracy as only those “on the inside” are allowed to take part in the discussions. The net effect is that a system intended to be scrutinized, that is predicated on the scientific use of animals, is not reviewed and scrutinized, but rather continuously consolidated [11,20,29,30,31]. Plous and Herzog [25] discussed how this results in an “ethical monoculture” that is likely to impair the ethical committees’ ability to meet public concern for research animal welfare. Another consequence is that the AECs, in practice, perform a technical or advisory role rather than actually making ethical judgments [32]. 

Varga [24] argues that it is valid to compose committees with members who represent different backgrounds and perspectives, as ethical issues are complex and society holds a diversity of views. Hansen, et al. [32] argues that because AECs are the only mechanism in place for addressing the ethics of animal research, it is vital that they consider major ethical issues to a greater extent than is done today. Therefore their composition also needs to reflect a greater ethical diversity than is currently the case in many countries [32] (Sweden is an exemption here). Further, it is crucial to understand the mechanisms behind ethical reflection by AECs as such, in order to improve the quality of the project evaluation. Hence, in the next section, we reflect on the origin of morality in relation to decision-making.

### 1.2. The Moral Emotion as the Origin of Morality

The origin of ethical norms and rules is an increasingly popular area of research, and the view that empathy is an important factor in ethical decision-making has grown in recent years [33,34]. Stone [35] argues that it is our ancient capacity for empathy that enables humans to be moral beings, and there is evidence suggesting that children’s moral values originate in the ability to empathize with others [36]. Goleman [37] says it is the ability to share another’s feeling of pain that makes a person wish to help or at least refrain from inflicting suffering on another. Thus, a person’s moral development is facilitated by the capacity to empathize, for example due to its role in determining our moral sensitivity, i.e., what we perceive as moral issues and to what extent [38].

Haidt [39] has developed what he calls ‘the social intuitionist model of moral judgment’, emphasizing that emotions play the most crucial role through the so-called gut feeling, which gives a quick, automatic, and often subconscious response, whereas the role of cognition/reasoning is to justify the initial response and aids in dealing with conflicting values and intuitions. For simplicity, empathy will herein however be referred to as an emotion although we are aware that research suggests that it is an ability that has both emotional and cognitive properties.

Mencl and May [40] studied how and to what extent decision-makers experienced that empathy affected their decisions. According to them, having an emotional attachment to the moral object, i.e., the object of moral concern influences both the recognition of a relevant moral issue and the intent to act morally. They further found that low emotional attachment and identification with the object, was related to higher focus on the outcomes rather than on the responsibilities of a situation, implying that a more utilitarian ethical approach is then expected [40]. This might well be understood as an attempt to achieve objectivity and, if it occurs in the context of AEC, also relates to the aim to make scientific decisions.

### 1.3. Scientific Norms and Emotions

Natural science is expected to deliver evidence-based objective results. Emotions are traditionally expected to be excluded, since they are seen as irrational and unreliable [41]. In line with this, the scientific community has perceived emotions as subjective, and therefore irrelevant [42]. However, apart from the fact that emotions are of crucial importance for our moral motivation, i.e., that scientists might well feel emotional engagement in solving their tasks, research suggests that the line between emotions and reason is not as clear as previously thought. In fact, these two constantly interact and together seem to form our moral judgments [37,39,41].

Galvin and Herzog [43] studied this in an AEC setting in 1992, and found important functions of, and differing views on, emotions in the review process. Some of their subjects strongly emphasized the role of emotions in their ethical judgments, while others expressed how they felt a need to separate emotions from rational reasoning, for fear that emotions would “disrupt reason”. The authors concluded that there may be ambiguous and value-laden views on emotions in the AEC setting. This was confirmed in the similar but more recent studies by Schuppli [26] and Ideland [20], where informants expressed that they strived to keep their emotions under control so that they did not interfere with rational arguments. 

According to Göthlin and Lantz [44], knowledge, rationality and emotionality are all required in order to make a well-considered ethical decision. Some people may be more inclined to one or two of these, for instance due to the culture of their profession [43]. In summary, seemingly conflicting perspectives exist on what is and should be guiding when it comes to our moral and ethical decision-making. Literature also indicates that the ethical harm–benefit model most commonly used might be flawed and too narrow to fit all relevant ethical aspects in AEC project evaluations [20,26,32,45]. We thus regard it worth considering in some detail how members of the AECs handle the often perceived incommensurability between reason and emotion, science and ethics, objectivity and subjectivity. This is a challenge in Swedish AECs where scientists and laypersons are supposed to participate equally in the decision-making process. Do they have the same opportunities to actively participate and influence the decision? Of particular interest are the member categories researchers and animal welfare laypersons, since they, according to abovementioned studies, have diverging views on a number of core issues. Yet, they need to reach a common decision on each single application.

### 1.4. Aims of the Study

We wanted to further investigate what role emotions such as empathy are allowed to play in the AEC decision-making process and if evidence of a conflict between reason and emotions can be found also in the Swedish committees. Focusing on traditional scientific norms, the harm–benefit assessment model in practice and the role of emotions, the specific aim was to investigate if members from different committee member categories differed. We hypothesized that there would be a difference between laypersons and researchers, with laypersons being more emotionally involved during the decision-making process. Further, we hypothesized that laypersons from animal welfare or animal rights organisations on the one hand and researchers on the other would have the most divergent views. The study was hence focused around three themes: the role of traditional scientific norms; problems of practicing the harm–benefit assessment model, and finally the role of emotions in decision-making.

## 2. Materials and Methods 

### 2.1. Quantitative Survey

The main part of the study was a web-based survey (carried out using the online service SurveyMonkey) (Appendix A) targeting all AEC members in Sweden in 2013. The questionnaire was piloted and reviewed by a group of ten persons with different experience and level of knowledge relevant to the topic, including researchers, former committee members, former research animal technicians, and animal welfare professionals. In total there were 182 Swedish AEC members in seven committees at the time but for reasons of confidentiality, invitations to participate in the study were sent by the committee secretaries via e-mail. Not all members used their e-mail regularly, and hence it was unknown how many members were actually reached. According to the secretaries, e-mail addresses were also lacking for a few people in each committee, and it was estimated that 161 committee members were reached by the invitation, including deputies and chairpersons. Responses were submitted anonymously online.

The questionnaire contained introductory general information, followed by questions addressing three main study topics: 1. Experience of work in the committee; 2. Perception and interpretation of the ethical decision-making process; and 3. Emotions in the decision-making process.

Most responses (7 of 10 questions) were supplied on a Likert scale from 1 to 5 [46], with 1 representing ‘completely disagree’ and 5 ‘completely agree’, while the remaining responses were supplied as yes/no or comments. Comparisons were made with regard to general experience of AEC meetings (1 test), the ethical decision-making process (5 tests) and emotions in the ethical decision-making process (4 tests). In each test, response scores from one or several questions were summed and the sum medians in different AEC member categories were compared using Mann-Whitney U (MWU) tests [47] in Minitab Express^®^ Statistical Software (Minitab Inc., State College, PA, USA). Clustering on AEC was disregarded, i.e., the answers of different respondents were considered as independent observations. *p*-values ≤ 0.05 were considered statistically significant.

### 2.2. Qualitative Interviews

In addition, and before the results from the questionnaire were analyzed, telephone interviews were conducted (by first author) with eight persons, representing different backgrounds and experiences from animal research and ethical evaluation in Sweden, as well as committee member categories, with the purpose of gaining a deepened insight into some of the key findings from the survey (Appendix B). The interviewees had not participated in the survey. Pseudonyms for participants were used in notes and interviews to ensure anonymity, and all participants gave informed consent to participation. The interviews were semi-structured with 10 initial questions centered on the three study topics. Interviews lasted 30 to 75 min and audio recordings were transcribed, validated by interviewees and translated from Swedish to English. These data were analyzed qualitatively using theoretical thematic analysis [48].

This research received no specific grant from any funding agency in the public, commercial, or non-profit sectors.

## 3. Results

### 3.1. Survey

In total, 80 persons responded to the survey but six had only responded to a few questions and were therefore removed. Of the 74 remaining responses, four were not fully complete but were included because more than two thirds of the questions were answered. This gives an estimated response rate of 46%.

Out of the 74 respondents, 41 (55.4%) were women and 33 (44.6%) men. The average age was 53 years, and most of the respondents had been committee members for 1 to 3 years (42%) or 4 to 6 years (33%). Eighteen percent had been members for more than 7 years. The respondents represented all member categories of the Swedish AECs: chairpersons (n = 2), researchers (29) or technical staff (6), laypersons from political parties (18), and laypersons from an animal welfare or animal rights organization (17); onwards called animal laypersons. Two respondents did not state category, but their responses were included in parts of the study where category was not an issue.

#### 3.1.1. Experience of Work in the Committees

**Figure 2:**
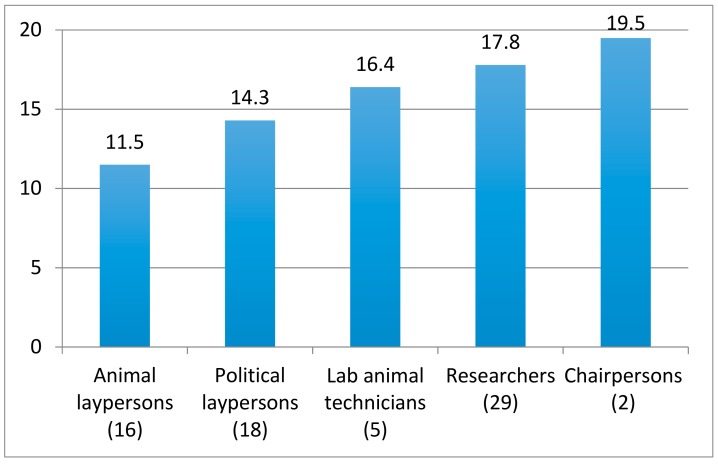
Animal Ethics Committee (AEC) members’ experience of plenary meetings. Sum scores for the following four statements: There is a good climate for discussion; I am being listened to; My contributions are taken seriously; I am able to influence the outcome. Each statement was scored from 1 (completely disagree) to 5 (completely agree).

There were clear differences between member categories in the general experience of AEC meetings (question 15a–d). Researchers and chairpersons had the most positive experiences while animal laypersons had the most negative ones (Figure 2), with a significant difference between researchers and animal laypersons (n_1_ = 29; median_1_ = 19; n_2_ = 16; median_2_ = 11; MWU = 832; *p* < 0.0001).

#### 3.1.2. The Ethical Decision-Making Process

Researchers and animal laypersons had quite different opinions on whether the AEC members interpreted ethical aspects of research on animals in the same way (82.4% of researchers and 50% of animal laypersons; question 20). Most researchers (82.1%) were of the opinion that all relevant aspects are considered in the ethical evaluation, while only 25% of the animal laypersons held this view (question 19). 

There were a few questions aiming to capture differences in ethical viewpoints, with researchers having higher scores than animal laypersons, for example, “All animal testing that can lead to any kind of benefit for humans is necessary and thereby justified” (question 21a; n_1_ = 28 median_1_ = 2; n_2_ = 17 median_2_ = 1; MWU 775; *p* = 0.001) and “I generally consider human suffering (physical and psychological) more important than that of animals” (21g; n_1_ = 28; median_1_ = 4; n_2_ = 17 median_2_ = 2; MWU = 811; *p* < 0.0001). For the statement “Mice and rats can suffer in a way which is comparable to humans”, researchers scored significantly lower than animal laypersons (21f; n_1_ = 28; median_1_ = 3; n_2_ = 17; median_2_ = 5; MWU = 512; *p* = 0.0015). When asking what aspects of a project evaluation the members thought contributed the most to the AEC decision, the three aspects deemed most important by all member categories were the purpose of the study, the potential suffering for the animals and the potential benefit to humans (questions 18 a, d and f). Researchers and animal laypersons had significantly different views on the actual importance of the first two of these aspects; animal laypersons to a lesser extent thought that the study purpose and the potential animal suffering were sufficiently considered in the evaluation process (18a; n_1_ = 28; median_1_ = 4; n_2_ = 17; median_2_ = 1; MWU 752; *p* = 0.0091 and 18d; n_1_ = 28; median_1_ = 5; n_2_ = 17; median_2_ = 3; MWU 785; *p* = 0.0004, respectively).

#### 3.1.3. Emotions in the Ethical Decision-Making Process

Laypersons and animal technicians stated that they were more emotionally engaged in the AEC evaluation, compared to respondents from the other member categories (Figure 2). Lab animal technicians and animal laypersons scored highest for experienced emotional involvement, i.e., the statement “I often get emotionally involved with my work in the AEC” (question 22a) and researchers scored significantly lower than animal laypersons (n_1_ = 26; median_1_ = 2; n_2_ = 17; median_2_ = 4; MWU = 460; *p* = 0.0043). Technicians and animal laypersons also to a higher extent answered that they often reacted negatively to the animals’ potential suffering, (question 22c) compared to the other member categories. Researchers and chairpersons scored lowest for this particular statement, with a statistically significant difference between researchers and animal laypersons (n_1_ = 26; median_1_ = 3; n_2_ = 17; median_2_ = 5; MWU = 413; *p* < 0.0001). There was however no significant difference between researchers and animal laypersons regarding the statement “I often react positively to the potential benefit of a study” (question 22b).

**Figure 3:**
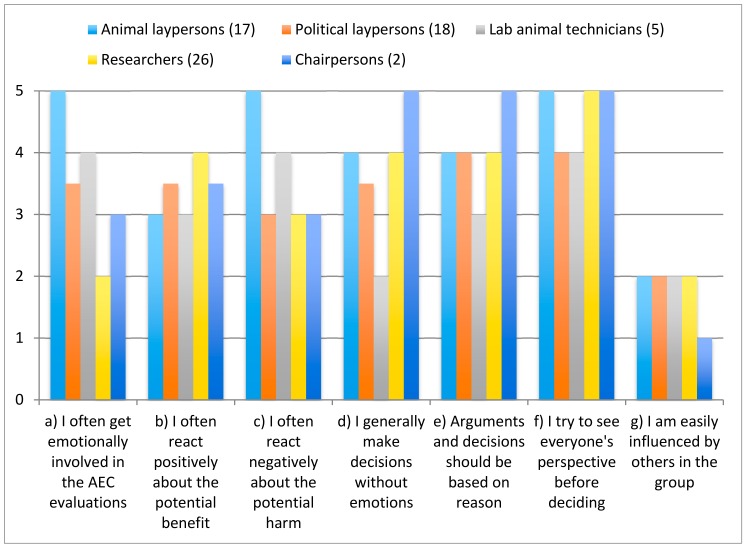
Aspects of emotional involvement in the AEC process. Statements were scored from 1 (completely disagree) to 5 (completely agree). See full statements in Appendix A, question 22.

Laypersons scoring highest on the statement “I often get emotionally involved in this task (question 22a) and “I often react negatively to potential suffering” (question 22c) also stated that they felt being the least influential in the decision process (Figure 3). However, there was no significant difference when comparing researchers and animal laypersons for the statements “I generally think that arguments and decisions should be based on reason rather than emotions” (question 22e) and “I generally make decisions without being influenced by my emotions” (question 22d). 

**Figure 4:**
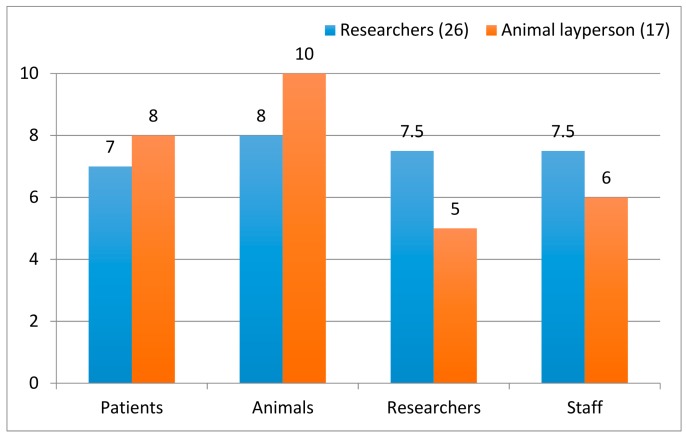
Researchers’ and animal layperson members’ combined median scores for statements in question 23 aiming to capture emotional and cognitive empathy for patients, animals, researchers and animal care staff. For every stakeholder two questions were included and scored from 1 (completely disagree) to 5 (completely agree), yielding a total maximum score of 10.

All respondents scored relatively high on questions aiming to capture affective and cognitive empathy in the decision-making process (question 23). In order to find out which was the AEC members’ core object of moral concern, they were asked with whom they primarily empathized in the research process assessed. They were also asked whether they most likely imagined being in the situation of people who might benefit from proposed studies (patients), the animals used, the researchers who apply for approval, or of the animal technicians who handled the animals. The respondents appeared to feel more or less strongly for these different objects of moral concern. Generally the animals seemed to evoke most empathy, followed by patients (Figure 4). Political laypersons scored highest for patients, researchers equally for them and the animals, while animal laypersons and lab animal technicians scored highest for the animals. The combined median for the statements “I feel for the animals that are used in the studies we evaluate” (23c) and “I imagine being in these animals’ situation” (23d) was significantly lower for researchers than for animal laypersons (n_1_ = 26; median = 8; n_2_ = 17; median = 10; MWU = 485; *p* = 0.0192). The opposite pattern was found for empathy towards the researchers whose applications were considered by the committees. Here, researchers scored significantly higher than animal laypersons (n_1_ = 26; median = 7.5; n_2_ = 17; median = 5; MWU = 661; *p* = 0.0264). There were no significant differences between researchers and animal laypersons regarding empathy for the patients or for the animal care staff.

### 3.2. Interviews

Four key themes were identified from the interviews and formed the basis of the analysis together with the quantitative results from the survey. The key themes were: (1) Personality and roles in the AEC affect the interplay and experience of working in an AEC; (2) Assumed level of knowledge gives advantage/precedence in the decision-making process; (3) Weighting of harm and benefit is difficult in practice and (4) Emotions have low status. As an example, Table 1 shows how the last of these themes was derived.

#### 3.2.1. Experience of Work in the Committees

Respondents expressed different views on their possibilities of influencing the discussion topic or decision associated with their different roles in the committees. A majority emphasized that there are certain requirements in order to be taken seriously, mainly that a certain personality or a perceived lack of knowledge or experience can lead to exclusion from the debate. The experience of the discussion climate seems to differ between member categories and also between committees. Some interviewees reported having experienced tensions, a harsh discussion climate and a feeling of exclusion from the discourse, while others thought that everybody had the freedom to express their opinions during the meetings. These differences were seen both between and within committees, and in general the same differences between member categories were found as in the survey, with animal laypersons having the least positive experience. Beside negative experiences of the discussion climate it was expressed in several interviews that because an assumed level of specific knowledge or higher education was required to be heard and considered credible, laypersons had a difficult position from the very beginning.

#### 3.2.2. The Ethical Decision-Making Process

Differing viewpoints on, for example, the primary object of moral concern and empathy (researcher, patient or animal) or what constitutes a proper ethical evaluation seemed to be a source of tension in some of the committees, especially when members wanted to emphasize different matters in the discussion. Further, the required harm–benefit analysis was difficult to carry out in practice, which created frustration for some committee members. Again, more knowledge about research design and expected impact on the animals would be needed in order to fully estimate potential harm and benefit. It was expressed that the discussions in the end focused more on technical improvements than ethical issues. 

#### 3.2.3. Emotions in the Ethical Decision-Making Process

The view on how emotions influenced the AECs’ evaluations appeared ambiguous. The majority of the respondents stressed that it was difficult to separate emotions from other considerations in the ethical evaluation, although at the same time they emphasized that “one should not let emotions take over, not least in order to gain support for one’s argument”. Several respondents expressed a fear to be considered “too emotional” and not being taken seriously if basing arguments on anything else than solely “hard facts”.

## 4. Discussion 

For a long time it has been debated whether reason or emotion should be our main guide to morality. According to Nussbaum [41,49] who in this case builds on Aristotle, the two perspectives are synergistic parallel processes that constantly interact with each other. A main function of our rational side is that it can help us realize when our ethical responsibility is lacking, and then help us reflect upon and improve our approach. Emotions, on the other hand, can be seen as “moral intuition”, which help us identify ethical issues that need to be further investigated. Based on this, we argue that both rational reflection and empathic abilities need to be part of a sound ethical discourse, also on issues in animal-based research. 

This study shows that most AEC committee members claimed to have quite a lot of empathy for those who were in different ways affected by the committee decisions. At the same time many respondents thought that decisions should be based on reason rather than emotion and stated in survey comments and interviews that emotions need to be kept “in place” in order not to dominate. In line with our hypothesis, animal laypersons and animal technicians expressed more emotional involvement in the AEC process than other member categories. The former groups, however, also expressed a feeling of being less influential and less content with the general committee work, compared with researchers. Researchers typically stated that emotions play a part in the ethical deliberations, but also that there is a risk for feelings to undermine reason. This indicates that researchers share a dualistic view on reason and emotion, and a preference for reason in line with traditional scientific norms. Hence, our study confirms the results of Galvin and Herzog [43] and Schuppli [26], which indicated that emotional and rational components interact to produce the decisions made by AEC members, but that there may be a conflict between them. 

According to Hoff [50] a problem with AECs (by reference to Luhmann’s systems theory) is that because the committees are derived from the scientific community, the prevailing norms will be of a scientific nature. This implies that all other norms and values will be subordinate to scientific ideals [11,50]. Emotions are traditionally not part of the scientific ideals, and there is a prevailing preference for reason-based arguments. As the animal laypersons get among the highest scores for emotional involvement in both the research animals’ situation and in the AEC discussions in general [26,43], we suggest that this, in combination with the experience of being less listened to if expressing emotions, could be a main reason for them to score the lowest on overall satisfaction with their AEC participation. 

We were also interested in problems associated with practicing the harm–benefit assessment. In the EU, this is the prescribed model for ethical evaluation of animal research, and our specific focus was on how the informants perceived this model in relation to, on the one hand, the outspoken scientific context and, on the other, wider ethical concerns not possible to cover in this model, such as animal integrity, animal rights and how to assess ethical values (see Ringblom, et al. [45] for the latter). 

Klein [28] suggests that the harm–benefit analysis prescribed in the AEC process is very different from how moral evaluations are normally made in other settings, where for example affect and intuition as well as imagination play crucial roles. Schuppli [26] concluded that committee members might not be aware of such differences, and that researchers might view the utilitarian model as the ‘only’ one. Further, our study confirms there is a perceived gap between the very basis of morality in empathy and emotional involvement, and the requirement of a harm–benefit analysis to exclude exactly these aspects. Also, ethical issues not related to measurable aspects, such as animal integrity or ethical values of life and death, seem difficult to include in the ethical evaluation. Improving AEC members’ insight into the width of ethical aspects could improve the quality of decisions [21,51]. We found that researchers and animal laypersons in particular appear to make fundamentally different ethical judgments, as well as hold disparate views on what are the relevant ethical aspects of the evaluations. This together with the fact that researchers are significantly more content with the functioning of the committees indicates that the ethical model used suits their preferences better. This is in line with findings by e.g., Schuppli [26], who reported that several community representatives/laypersons on Canadian AECs found the atmosphere intimidating and that common problems were perceived difficulties of being an ‘outsider’ or to feel that their contribution was not appreciated. As the laypersons seem to make other moral judgments while at the same time feeling less influential, one can question whether or not the ethical evaluation that takes place is open enough to consider the variety of participants’ arguments and contributions. 

There are many factors affecting ethical decision-making, such as personality, knowledge, gender, social hierarchies, relationship to the objects of moral concern, actual context and group dynamics, personal preferences, professional roles, and experiences [40,52,53,54,55]. One can see a paradox in aiming for ethical decisions to be strictly logical as in utilitarian or deontological ethics which are “scientific or objective by nature” while at the same time the preceding choice of ethical theory and the ethical thinking as such are strongly influenced by the value system of each individual [53]. It is however clear from the literature that social relations also to a large extent affect decisions [56]. For example, the more people in a group share a particular identity, the more likely polarization and suppression of opposite viewpoints occur [57]. Consequently, the less alone a person feels in his or her opinion the easier it is to stick to it [58]. In a group setting such as an AEC there is also a cost to have a different opinion to an otherwise homogenous group as this can easily cause emotional distress [59]. This might be especially true if the feeling is that one’s opinion is not taken into account anyway. Animal laypersons appear to have views that differ from the scientist members, and animal laypersons are at the same time the least content with the AEC process. According to Hansen, et al. [32], there is however a significant risk that relationships among researchers, veterinarians, and other institutional faculty and staff who comprise the majority of AECs can create an environment where committee members do not want to criticize their peers’ work. 

One of the best ways to diminish judgment bias is to include multiple perspectives in the decision-making process [32]. Judgments should preferably be independent so as not to share any bias but this can of course be difficult to achieve in practice. Efforts should be made to welcome different ethical views into the discussion to enable a wide variety of views as the task of the committee is to make decisions on behalf of society. It thus seems crucial that there is an equal distribution of member categories in the ethical committees and that a respectful and open atmosphere is provided, where all committee members feel valued and not intimidated, as argued by Schuppli and Fraser [56]. They suggest specific training about roles in the AECs, especially clarifying the role of non-scientist members. Also, the chairperson’s style of leadership is of utmost importance to encourage an inclusive and balanced and yet honest dialogue and evaluation (Lo, 1987). We therefore agree with Schuppli and Fraser [56] that training of chairpersons to ensure all members feel their views are taken into account will most likely improve the function of the committees to a large extent, although this alone will not be enough. 

Habermas [60] formulated the probably most well-known method for handling social hierarchies in decision-making situations, aiming at mutual understanding and an ethically justified decision. He suggests that if a certain set of discussion rules are established and followed, the “ideal speech” situation can be achieved: Every subject with the competence to speak and act is allowed to take part in the discourse; everybody is allowed to question any assertion, to introduce any assertion into the discourse and to express his or her attitudes, desires and needs, and finally, no speaker may be prevented from exercising his or her rights laid down by the above principles [60]. These, or similar, discussion rules could be used in order to improve the ethical discussion as well as the discourse climate of the AECs [7,61,62]. Applying such discourse rules for decision-making in the AECs could contribute to ensuring respect for each participant’s view and argument, as well as break down the hierarchical structure to which many laypersons testify. Not until they feel equal in the discussion can they truly fulfil their task as society’s representatives. Such a decision-making structure could facilitate the conversion of the AEC review process into a proper evaluation of ethically relevant aspects and consequently better meet the requirements of the EU Directive.

Finally, this study aimed to investigate if emotions affect ethical deliberations in AECs. Emotions such as empathy have an important role to play in decision-making, not least for the motivation to actively participate in the discourse, but also since it is inevitable to form moral judgments at least to some extent based on what we initially perceive as right or wrong [39]. In our study, a majority of respondents stated that they empathized with the different groups affected by the committee’s evaluation. We could also see that the committee members had most empathy towards those whose interest they mainly aimed to safeguard. Researchers were for example significantly more empathic towards researchers who sought ethical approval, while animal laypersons were significantly more empathic towards the research animals. Considering the inherent and inevitable effect of emotions in general, and empathy in particular, on our morality and in ethical deliberations, the expressions of these dimensions need to be allowed in ethical decision-making. Such dimensions are included in the tradition of ethics of care, which is often based on the feminist ethics’ critique of the principle-based ethical theories utilitarianism and deontology. According to ethics of care the relationship as such has moral significance, and promotes the care of the individuals (human or non-human) one is responsible for. In line with this, interdependence between sentient beings and focus on the actual situation are core elements in this ethical discernment within ethics of care [63]. One limitation of this theory of course lies in the fact that some individuals exist outside a personal relationship, but in the case of animals used for research this will not be a relevant critique [21]. Rather, an ethics of care can facilitate understanding and handling of the empathy experienced by member categories towards researchers, patients and animals.

According to de Waal [64] our level of empathy is affected by whether we can socially afford to be affected, as well as whether we want to be. Many AEC members reported how they strived to keep emotions in check and expressed that arguments are commonly dismissed simply for being considered “emotional”. This likely leads to a problematic discrepancy, not least for the animal laypersons and animal technicians who express being more emotionally involved in the AEC process. If empathy is a natural part of how we make moral judgments, it appears paradoxical that it should be disregarded due to prevailing scientific norms and the fact that the applied ethical model is unable to encompass it.

## 5. Conclusions

Based on the findings of our study, it seems relevant to make a clear distinction between scientific and ethical reasoning in the AECs. These two perspectives can be seen as two languages that meet and by which we try to evaluate the moral justifications of animal research. Perhaps a way forward would be to start questioning utilitarianism as the only and superior ethical model in the AEC context, as it is based on certain flawed assumptions of objectivity in ethical discernment, such as that interests of animals and humans have equal importance, that all necessary knowledge is equally accessible for committee members, and that there is an objective estimate of potential harms and benefits. The different roles of the different committee member categories should also be clarified, in order to better acknowledge the advantage of them bringing different perspectives to the table.

Furthermore, due to emotions such as empathy being the very basis for morality, it appears illogical to actively disregard it in moral deliberations. Instead we should aim for a balanced and more inclusive way of making ethical decisions, which is more likely to appeal to people both inside and outside the scientific community. A more balanced project evaluation can also be achieved by acknowledging factors such as group dynamics and social hierarchies. Applying discourse rules for decision-making, inspired by e.g., Habermas, might be one way forward.

## Figures and Tables

**Table 1:** Example of how interview themes were derived through thematic analysis.

Condensed Meaning Unit	Interpretation of Meaning (Codes)	Theme
The more unemotional you are the more you are listened to	Experience that expressing emotions is unsuitable	Low status of emotions
She was emotional which made it easier to dismiss her		
Those who are empathic get depressed since there is no use		
It is difficult to involve emotions since everything is very abstract	Rational reasoning is favored	
Arguments based on facts generally gain more support		
Arguments were generally quite harsh		
One should be professional enough to not let one’s emotions matter	Emotions are seen as irrelevant	
It is not that we sit and feel sorry for the animals		

## Supplementary Materials

The following are available online at http://www.mdpi.com/2076-2615/8/10/181/s1.

## Appendix A. Survey

A survey about decision-making in Animal Ethics Committees (AECs)

General

1. I am: Woman Man

2. Year of birth:

Questions about your role in the AEC

3. I am a member of the following committee: _________

4. Experience as committee member: <1 year, 1–3 years, 4–6 years, 7–9 years, >10 years

5. What role do you have in the committee? I am a: Scientist, Laboratory animal technician, Layperson from a political party, Layperson from an animal welfare or animal rights organization, Chairperson or deputy chairperson

Pick those options that describe you best

6. I have studied the following topics at university (also shorter courses): Medicine, Veterinary Medicine, Biology, Ethology, Laboratory Animal Science, Animal Welfare, Animal Care, Philosophy, Ethics, Theology, Law

7. I have studied the following topics elsewhere: Medicine, Veterinary Medicine, Biology, Ethology, Laboratory Animal Science, Animal Welfare, Animal Care, Philosophy, Ethics, Theology, Law

8. When I started as committee member I had training related to ethical evaluation: Yes No

9. I have attended training for AEC members organized by the Swedish Board of Agriculture: Yes No

10. I feel that I have sufficient scientific knowledge for my task in the AEC: Yes No

11. I feel that I have sufficient knowledge about ethics for my task in the AEC: Yes No

12. Multiple interests can be said to be represented in the committees. I see myself as primarily representing (one or more options): Science, the public, the patients who might benefit from the research, the animals, other: _____

13. Comments on this section:

14. The following statements concern your experience of the panel group meetings. Choose the number between 1 and 5 that best describes to what extent you disagree or agree for the following statements:

Completely disagree (1) Completely agree (5)

a. There is a good climate for discussion

b. I am being listened to

c. My contributions are taken seriously

d. I am able to influence the outcome

e. The discussions are relevant

f. There is enough space for ethical discussions

g. Every application is given sufficient time

15. The following statements concern your experience of the plenary meetings. Choose the number between 1 and 5 that best describes to what extent you disagree or agree for the following statements:

Completely disagree (1) Completely agree (5)

a. There is a good climate for discussion

b. I am being listened to

c. My contributions are taken seriously

d. I am able to influence the outcome

e. The discussions are relevant

f. There is enough space for ethical discussions

g. Every application is given sufficient time

16. Comments on this section:

Questions regarding the ethical evaluation

17. Choose the number between 1 and 5 that best describes to what extent you disagree or agree for the following statements:

Completely disagree (1) Completely Agree (5)

a. I feel certain as to how to interpret what benefit can be considered “valid in the view of the public” (wording from the Swedish Animal Welfare Act)

b. I feel certain as to how to decide whether or not alternative methods to animal testing could be used as replacement

c. I feel certain as to how to decide whether or not the proposed methods could be refined

d. I feel certain as to how to decide whether or not the suggested number of animals is reasonable

e. I feel certain as to how to evaluate whether or not the potential suffering is greater than what is “absolutely necessary” (wording from the Swedish Animal Welfare Act)

f. I feel certain as to how to evaluate what is a reasonable end point

18. How much do you think the following aspects of an application affects the evaluation?

To a very small extent (1) To a very large extent (5)

a. The purpose of the study

b. The number of animals that will be used

c. Which animal species will be used

d. The potential suffering for the animals

e. The scientific quality of the study

f. The potential benefit to humans

g. The potential benefit to animals

h. The potential benefit to the environment

i. The options for alternative methods

j. Whether or not the study is categorized as of small, moderate, considerable, or terminal difficulty

Comments:

19. I feel like all the previous aspects are being considered during the AEC meetings: Yes No

20. I feel like all us committee members have similar views on what are the ethical aspects of animal testing: Yes No

Comments:

21. When I am weighing the animals’ potential suffering against the potential benefits, I generally have the following outlook. Choose the number between 1 and 5 that best describes to what extent you disagree or agree for the following statements:

Completely disagree (1) Completely agree (5)

a. All animal testing that can lead to any kind of benefit to humans is necessary and thereby justified

b. All animal testing that can lead to curing human diseases is necessary and thereby justified

c. All animal testing that is not directly about finding cures for human diseases but that instead can be considered ’basic research’ is necessary and thereby justified

d. Only animal testing that can benefit animals is necessary and thereby justified

e. Only animal testing that can benefit the environment is necessary and thereby justified

f. Mice and rats can suffer in a comparable way to humans

g. I generally consider human suffering (physical and psychological) more important than that of animals

h. I generally consider human and animal suffering to be of equal importance

Comments:

Questions about the role of our emotions in ethical decision-making

22. Choose the number between 1 and 5 that best represents to what extent you agree or disagree for the following statements:

Completely disagree (1) Completely agree (5)

a. I often get emotionally involved with my work in the AEC

b. I often react positively about the potential benefit of a study

c. I often react negatively about the potential animal suffering

d. I generally make decisions without being influenced by my emotions

e. I generally think that arguments and decisions should be based on reason rather than emotions

f. I try to see things from everyone’s perspective before making a decision

g. My emotions are easily influenced by the atmosphere and by the other committee members

23. Choose the number between 1 and 5 that best represents to what extent you agree or disagree for the following statements:

Completely disagree (1) Completely agree (5)

a. I feel for the people that might benefit from the studies we evaluate

b. I imagine being in these people’s (patients) situation

c. I feel for the animals that are used in the studies we evaluate

d. I imagine being in these animals’ situation

e. I feel for the researchers that apply for ethical approval

f. I imagine being in these researchers’ situation

g. I feel for the staff that carry out animal testing procedures

h. I imagine being in these people’s (animal care staff) situation

Comments:

24. Suggestions about how the ethical evaluation can be improved:

25. Feedback on this survey:

## Appendix B. Interview Questions

1. Animal testing is perhaps the one area of animal use that is most developed in terms of mandated ethical evaluation, why do you think this is?

2. Which do you think is the most important function of the ethical evaluation?

3. Which do you think are the most important and the most difficult ethical issue respectively regarding animal testing?

4. What knowledge to you think is most important in order to be able to carry out the ethical evaluation?

5a. What is the ethical evaluation supposed to be based on according to you?

b. Is this in accordance with what you think it should be based on?

c. Is this clear to the ones who are supposed to carry out the evaluation?

d. Can there be different interpretations?

e. How is the harm–benefit analysis carried out in practice in your committee?

f. Is there any aspect in particular that you think seems most decisive, that outmost affect the decisions?

6a. How do you experience your role as … in the committee?

b. Do you consider yourself representing any particular interest?

7a. How does the committee handle different interests and outlooks in the discussions?

b. How do you think this should be handled?

8a. What view is there on emotions/emotionality in the committee?

b. To what extent do you think that emotionally led aspects such as empathy or lack thereof influences the decisions?

9. How do you think the ethical evaluation works today?

10. Is there anything you think ought to be improved?
